# Case Report: Uterine Adenosarcoma With Sarcomatous Overgrowth and Malignant Heterologous Elements

**DOI:** 10.3389/fmed.2021.819141

**Published:** 2022-01-10

**Authors:** Yunuén I. García-Mendoza, Mario Murguia-Perez, Aldo I. Galván-Linares, Saulo Mendoza-Ramírez, Norma L. García-Salinas, Julio G. Moctezuma-Ramírez, Blanca O. Murillo-Ortiz, Luis Jonathan Bueno-Rosario, Marco A. Olvera-Olvera, Guillermo E. Corredor-Alonso

**Affiliations:** ^1^Department of Surgical Pathology, Speciality Hospital N°1 National Medical Center Bajio, Social Security Mexican Institute, University of Guanajuato, Leon, Mexico; ^2^Head of Department of Surgical Pathology, Speciality Hospital N°1 National Medical Center Bajio, Social Security Mexican Institute, University of Guanajuato, León, Mexico; ^3^Pathology Unit, General Hospital of Mexico “Dr. Eduardo Liceaga”, National Autonomous University of Mexico, México, Mexico; ^4^Research Unit of Clinic Epidemiology, Speciality Hospital N°1 National Medical Center Bajio, Social Security Mexican Institute, Leon, Mexico; ^5^Head of Oncology Unit, Speciality Hospital N°1 National Medical Center Bajio, Social Security Mexican Institute, Leon, Mexico; ^6^Department of Radiology, Speciality Hospital N° 1 National Medical Center Bajio, Social Security Mexican Institute, University of Guanajuato, Leon, Mexico; ^7^Department of Pathology, General Hospital 92, Social Security Mexican Institute, Acuña, Mexico

**Keywords:** uterine adenosarcoma, heterologous elements, immunohistochemistry, uterine sarcoma classification, malignant neoplasm

## Abstract

A 46- year-old woman presented a uterine adenosarcoma originating in the lower uterine segment. The diagnosis was made in an endometrial biopsy and confirmed in the pathological examination of the complete surgical specimen, both identifying heterologous malignant elements. In addition, complementary immunohistochemical studies were performed. We reviewed the literature, illustrating the clinical and morphological characteristics and the differential diagnoses to be evaluated.

## Introduction

Endometrial cancer corresponds to the vast majority of malignant tumors of the uterine corpus (80% in Europe and more than 90% in the United States). It is the sixth most diagnosed cancer in women and the second most exclusively diagnosed female genital tract cancer (1). Uterine adenosarcoma (UA) is a mixed tumor of the uterus consisting of a benign glandular epithelium and a malignant mesenchymal component, which was reported as müllerian adenosarcoma by Clement and Scully in 1974 (2, 3). It is a rare tumor that corresponds to 8% of all uterine sarcomas and <0.5% of uterine malignant tumors (4, 5). The morphological characteristics of these neoplasms are distinguished from other biphasic tumors (epithelial and mesenchymal), both benign and malignant. UA are generally neoplasms of low malignant potential, except when accompanied by sarcomatous overgrowth and myometrial invasion (1, 4); these tumors can recur locally, and rarely metastasize (6).

About 15% of UA can have heterologous elements, such as skeletal muscle, cartilage, fat, and other components. Such heterologous elements are less common than the homologous variant (7). We present a case of uterine adenosarcoma with sarcomatous overgrowth (UASO) with heterologous elements in a middle-aged woman, and we review its clinical-pathological characteristics and management.

## Case Description

A 48-year-old woman with a gynecological-obstetric history of 2 pregnancies, obtained by cesarean section. Her condition began 6 months before admission, with intermittent metrorrhagia, dyspepsia, pain; increased abdominal volume and metrorragia were added. A Colposcopy and biopsy was performed, which reported a high-grade endometrial stromal sarcoma vs. carcinosarcoma. Subsequent symptoms were presented; abundant transvaginal bleeding and evidence of hypovolemic shock, requiring a transfusion of 3 blood packs and a hemostatic radiotherapy dose of 6 Gy. A revision of previous pathological material was requested to establish therapeutic conduct.

## Diagnostic assessment

**Figure 1:**
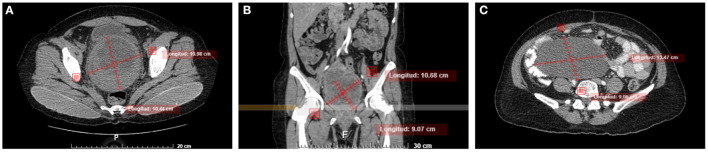
Abdominal axial **(A)** and coronal **(B)** non-enhanced CT images show a large well-defined rounded tumor, with a heterogeneous appearance in the cervix and demonstrating mass effect in adjacent structures and preserving interface with the bladder wall and rectum. **(C)** Axial non-enhanced CT, three months after surgery shows a heterogeneous abdominal tumor that compresses and displaces adjacent structures.

The slide review revealed a mixed malignant neoplasm consisting mostly of spindle-shaped and ovoid stromal cells arranged in a tapered pattern, with pale eosinophilic cytoplasm, nuclei with dense chromatin, evident nucleoli, and atypical mitoses; They coexisted with glands with an endometrial, tubular, and semitortuous appearance, lined by columnar epithelium with a proliferative appearance, with pseudostratified, discrete atypia, almost absent, without mitosis and luminal secretion. Around these glands, we observed packing of the spindle cells, arranged in a string of pearls pattern, and toward the periphery, there were paucicellular areas. The formation of “leaf-shaped” structures was also identified, with a low columnar epithelial lining, without atypia, and below it, the elongated cells described above. Extensive areas of highly cellular cartilage were identified, with moderate pleomorphism, hyperchromasia, and evident nucleoli. No areas of necrosis were identified. It was reported as UASO with heterologous elements of grade 2 chondrosarcoma (Figure 1).

**Figure 2:**
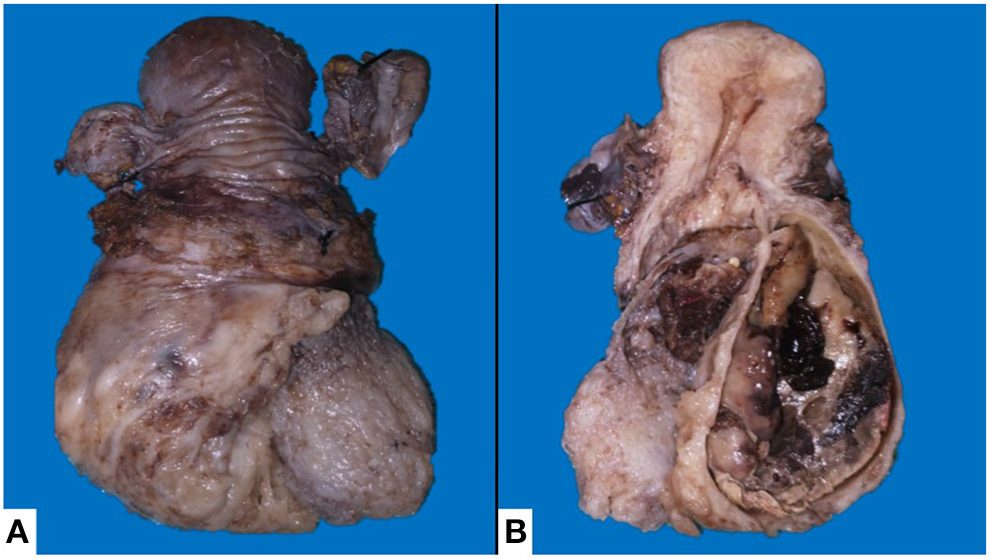
Macroscopic surgical specimen. **(A)** External surface of the posterior aspect of the uterus, a lesion that completely distorts the architecture of the cervix can be seen, the lesion has a pseudo-encapsulated fleshy appearance with some areas of irregular myxoid appearance. **(B)** Uterus, cut surface, with tumor originating in the lower uterine segment, with protrusion through the endocervical canal and expansion of the exocervix, showing the involvement of the endocervical wall.

A computed tomography scan was performed that showed a neoformative process at the cervical level (Figures 2A,B). An abdominal ultrasound reported a uterine morphological alteration, at the level of the isthmus with an oval nodule with poorly defined margins, with internal vascularity, the endometrium with homogeneous echogenicity, and without alterations in the ovaries. With these findings, she was scheduled for a total abdominal hysterectomy with bilateral salpingo-oophorectomy and appendectomy.

**Figure 3:**
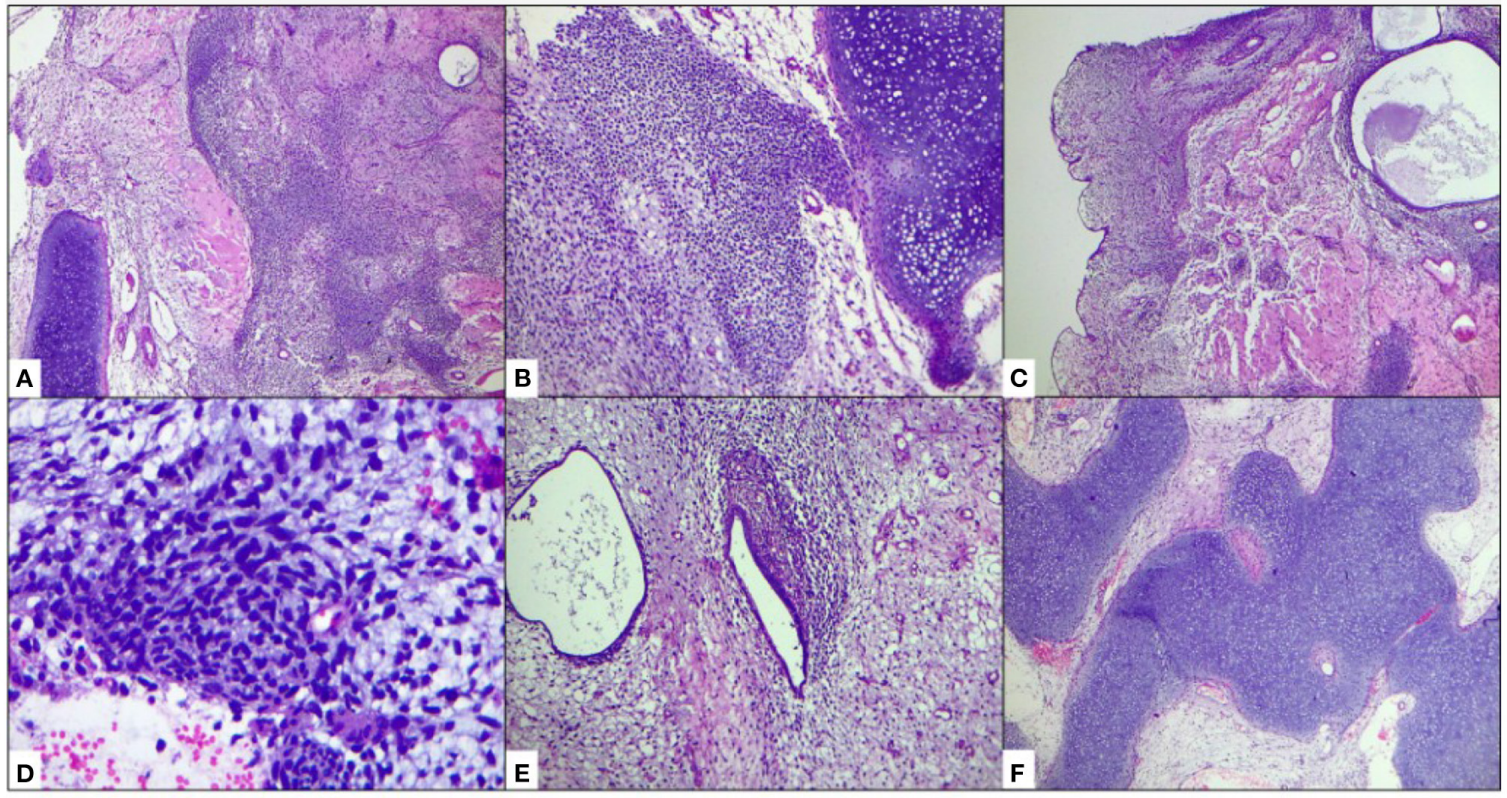
Histology of the tumor. **(A,B)** A mixed malignant neoplasm made up of cartilaginous areas that alternate with areas of paucicellular spindle malignant cells is observed, within which tubular and ectatic glandular structures can be seen. **(C)** Formation of “leaf-shaped” structures. **(D)** Malignant high-grade spindle cell component. **(E)** Glands with a “benign” endometrial appearance, surrounded by packed malignant stromal cells (cambium layer). **(F)** Extensive areas with malignant chondroid differentiation.

**Figure 4:**
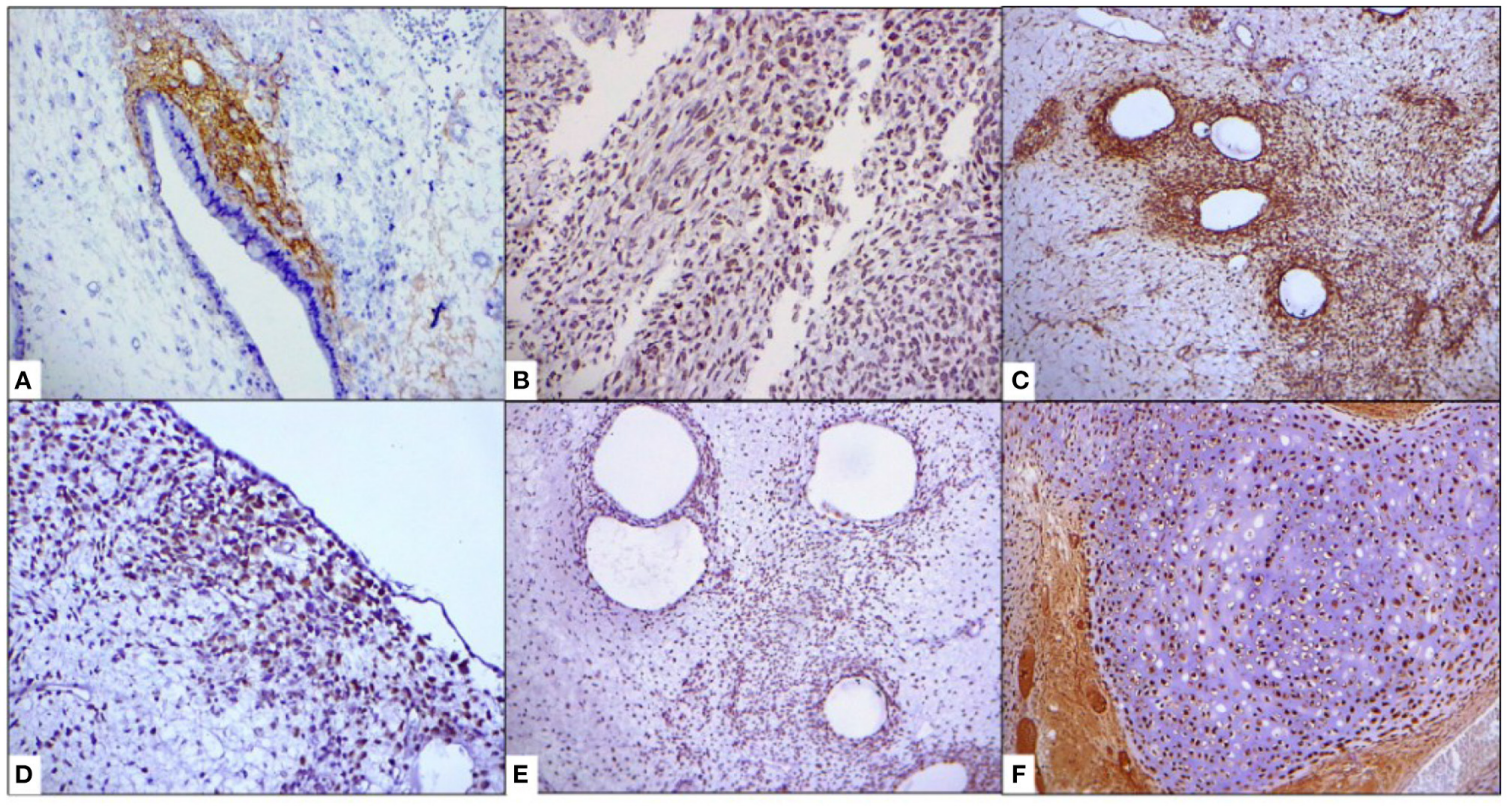
Immunohistochemistry. **(A)** CD10. **(B)** p53. **(C)** Beta-catenina. **(D)** hTERT. **(E)** PMS2. **(F)** S-100.

We received in our pathological anatomy department a surgical specimen that included cervical, endocervical, and lower uterine segment deformity, identifying an irregular tumor located toward the left inferolateral portion. We observed a heterogeneous tumor originating in the lower uterine segment, 9 cm in diameter, friable, grayish-brown, with hemorrhagic areas and areas of cartilaginous appearance, protruding through the endocervical canal, with expansion to the exocervix, without ulceration of the epithelium. No involvement of the endometrium and myometrium of the fundus and uterine walls were identified; however, there is 100% invasion of the left muscular wall of the lower uterine segment (Myometrium of the lower uterine segment thickness: 1.7 cm. Tumor size: 9 cm) (Figure 3). The rest of the organs sent did not present macroscopic alterations. The histological sections showed what was previously observed, direct involvement of the left parametrium of endometrial stromal sarcoma and chondrosarcoma component, without vascular invasion. Surgical margins were negative. Immunohistochemistry was positive for CD10, estrogen and progesterone receptors, p53, cyclin D1, WT1, PMS2, b-catenin an S100, it last found chondrosarcoma component (Figure 4). The final diagnosis was UASO with heterologous elements of grade 2 chondrosarcoma, with extension toward the endocervix and protrusion of the endocervical canal (pT1C, FIGO IB).

The patient was discharged and was kept under active surveillance by the oncology service, and without receiving adjuvant therapy. However, three months after the surgery, the patient presented intermittent abdominal pain. Abdominopelvic computed tomography was performed identifying a tumor adhered to the pelvic and abdominal cavity with dimensions of 30 × 25 cm (Figure 1C), with multiples peritoneal implants, for which she was operated on again. The patient underwent optimal debulking plus packaging, presented bleeding of 5,000 ml, 4 globular units and expander fluids were administered. We received in the Pathology department, a multifragmented tumor with a soft necrohemorrhagic appearance, and in the histological study with the characteristics already mentioned above. During her hospitalization, she developed pulmonary embolism. A pulmonary embolism computed tomography was performed, where a thrombus was evidenced in the right pulmonary artery, which led to a pulmonary infarction of the right lower lobe. Acenocoumarin was administered, despite this, she developed right lower extremity thrombosis. It was assessed by the Departments of Clinical Oncology and Radiotherapy, concluding that it was out of clinical treatment based on the added underlying pathologies. He is currently in palliative treatment, with acenocoumarin and morphine for pain management.

## Discussion

Mixed uterine tumors with an epithelial and mesenchymal component comprise a heterogeneous group of neoplasms that are categorized as carcinosarcoma, adenosarcoma, adenofibroma, adenomyoma, and atypical polypoid adenomyoma, included in the 2020 World Health Organization (WHO) classification (1, 8). The WHO defines a uterine adenosarcoma (UA) as a mixed epithelial and mesenchymal tumor where the epithelial component is benign or atypical, and the stromal component has a low malignant potential (1). They are rare neoplasms and constitute the third most common subtype of uterine sarcoma, after leiomyosarcoma (LMS) and low-grade endometrial stromal sarcoma (LG-ESS). Of the uterine malignant tumors, it corresponds to <0.5% of the cases (4, 5), and 8% of the uterine sarcomas. Most occur in postmenopausal women, but about 30% of premenopausal women, including adolescents. The average age of presentation is 58 years (range 50–59 years) (1, 9). Symptoms are non-specific, although the most common is abnormal uterine bleeding (4). Other symptoms that may occur are an enlarged uterus, protrusion through the cervical canal, and pelvic pain, among others. The most characteristic symptom is a recurrent polypoid lesion, which is biopsied on several occasions, in which a definitive diagnosis cannot be reached; A misdiagnosis can even occur, of which the most frequent is a cervical polyp (10). These tumors can occasionally occur outside the uterus (ovary and fallopian tube) (11).

The pathogenesis of UA is uncertain. However, the association between exogenous hormone therapy and the development of malignant tumors in endometriosis is well-known, and many studies have suggested the possibility of neoplastic transformation at endometriotic sites (10). Macroscopically, they are polypoid or multipolypoid lesions that can potentially occupy the entire uterine cavity and protrude through the endocervical orifice. They generally begin in the lateral walls of the uterine fundus, but they can also originate in the lower uterine segment; depending on its growth, it can affect neighboring structures (1, 8). Microscopically, it is a mixed lesion, with a “benign” glandular component immersed in a low-grade sarcomatous stroma (1, 8, 10). The glandular component appears inactive, and may occasionally present hyperplastic features with or without atypia, or, as in our case, a proliferative in appearance. Occasionally other types of the epithelium (mucinous, squamous, secretory, or clear cells) may be found. Glands are often seen in the myometrial invasion areas of uterine adenocarcinoma, suggesting that, despite its inactive glandular appearance, this component is an active part of the neoplasm (1, 2, 4). The stromal component is the most striking feature of these tumors, in which a low-grade sarcoma with variable cytological atypia (generally low to moderate) is seen. One of the diagnostic keys of this entity is the densification of the stromal component around the glands, surrounding them like a collar (it is sometimes called in some texts “cambium layer”), identifying mitoses, which are not >4 per field at 40X. The stromal component also presents a paucicellular component below the superficial epithelium, giving a “leaf appearance.” The anchoring of the uterine wall is usually well-defined; however, myometrial invasion is occasionally seen, which is related to a worse prognosis. Areas of heterologous differentiation (1, 8, 10) (skeletal muscle, lipoblasts, cartilage, etc.), or of stromal overgrowth (which is defined as a sarcomatous component that occupies 25% or more of the total tumor volume) (2, 10). Heterologous components can be observed in the last cases, especially rhabdomyosarcoma, and this represents a more aggressive clinical course. In one of the longest series published (7 cases), none of the cases with sarcomatous overgrowth presented heterologous areas (11). Our case presented a heterologous component of grade 2 chondrosarcoma, which is exceptional and has not been previously reported in the literature. UA that are considered high-grade have a high mitotic index (more than 4 mitoses per field at 40X) and are aneuploid with a fraction in the synthesis phase >10% (12, 13). In these cases, DICER1 gene mutations have also been observed, and less frequently, somatic alterations such as aberrations in the RAS or PI3K/PTEN signaling pathways, CDK4/MDM2 amplification, and TP53 and ARID1A mutations (14).

Due to its rarity, information on immunohistochemical markers that may be useful for the diagnosis of the entity is limited. Similar to endometrial stromal sarcoma, they are positive tumors for CD10, WT1, estrogen receptors, and progesterone. In cases with sarcomatous overgrowth, the expression of these markers is lower, which reflects the differentiation of the mesenchymal component (10, 15). In a paper presented at the USCAP 2018 conference, Stout et al. found immunoexpression of PTEN (70%), TERT (90%), PMS2 (100%), and Beta-catenin (60%); compared with low-grade endometrial stromal sarcoma, the latter also expresses the aforementioned antibodies in variable percentages, but is distinguished by finding rearrangement of the JAZF1 gene by fluorescent *in situ* hybridization (FISH), not being found in any case in UA; in our case, in addition to the traditional markers, we found expression of PMS2, Beta-catenin, and h-TERT. Alterations in the TP53 signaling pathway have been reported to be related to nuclear atypia and marked pleomorphism identified at lower magnification; Furthermore, the expression of the p53 protein by immunohistochemistry correlates mostly with the mutation of the gene (16). We clarify that the case was not studied molecularly, firstly because we do not have access to molecular techniques to study the tumor, and secondly because it does not provide greater benefit to choose medical therapy.

The differential diagnosis is broad and consists mainly of tumors that present a biphasic pattern, and vary depending on the type of adenosarcoma to be considered. In low-grade UA, endometrial polyps with unusual characteristics, adenofibromas, adenomyoma, and atypical polypoid adenomyoma are considered. In the spectrum of UASO, it is mainly with carcinosarcoma, although undifferentiated uterine sarcoma, endometrial stromal sarcoma, and leiomyosarcoma must also be considered (1, 10, 12).

The treatment of choice is surgical, performing total abdominal hysterectomy or by laparoscopic approach, with or without bilateral salpingo-oophorectomy; in women of reproductive age, a myomectomy and polypectomy may be chosen. There is no standardized chemotherapy, hormonal therapy, or radiation therapy in UA, due to the small number of patients with this neoplasia; however, standardized chemotherapy for uterine sarcomas, such as doxorubicin, ifosfamide, or gemcitabine/docetaxel, and newer drugs, such as trabectedin, appear to have some efficacy in UASO (16, 17).

The prognosis of UA depends on the stage and the presence of sarcomatous overgrowth. It has been observed that a 2-year survival and progression-free rates for patients with low-grade UA were 100%, compared to 20% found in those with UASO. Survival rates range from 60% for tumors with myoinvasion, and <50% present metastasis. The pelvis or abdominal cavity is the first site of recurrence, and sarcomatosis is a frequent complication; distant metastases are less common, with the liver and lung being the most frequent sites, followed by bone, kidney, spleen, and rarely, brain (1, 10, 16).

Due to the low incidence and histological diversity of uterine adenosarcoma, only a few case reports and series provide data on prognostic factors and survival prediction (18). With the rapid development of artificial intelligence, a new choice is provided for adenosarcoma researchers. The deep learning method has the ability to analyze the non-linear correlations that are more common in the real world. It has been proven to be greatly effective for various clinical tasks, including image identification, pathological diagnoses, genomic analysis, metabolomics, and immunology studies. Qu et al. developed and validated a personalized survival prediction model for UA, combining the SEER database with a neural network model called “neural multitask logistic regression model” (N-MTLR), comparing it with the traditional model (CPH model), demonstrating survival time predictions that are much more accurate than the other model (19). It is possible that the extension of this model in an online program, in case of obtaining data from cases around the world, could offer more information about this rare tumor.

## Data Availability Statement

The original contributions presented in the study are included in the article/supplementary materials, further inquiries can be directed to the corresponding author/s.

## Author Contributions

MM-P: conceptualization, supervision, writing original draft, and project administration. YG-M: investigation, writing original draft, and software. AG-L: resources and data curation. SM-R: resources and methodology. NG-S, JM-R, BM-O, MO-O, and GC-A: resources. LB-R: visualization. All authors contributed to the article and approved the submitted version.

## Conflict of Interest

The authors declare that the research was conducted in the absence of any commercial or financial relationships that could be construed as a potential conflict of interest.

## Publisher's Note

All claims expressed in this article are solely those of the authors and do not necessarily represent those of their affiliated organizations, or those of the publisher, the editors and the reviewers. Any product that may be evaluated in this article, or claim that may be made by its manufacturer, is not guaranteed or endorsed by the publisher.
